# From function to wellbeing: healing design strategies in contemporary outdoor fashion

**DOI:** 10.3389/fpsyg.2025.1687270

**Published:** 2025-12-02

**Authors:** Yijing Li, Seonju Kam

**Affiliations:** Department of Clothing and Textiles, Kyung Hee University, Seoul, Republic of Korea

**Keywords:** wellbeing, healing design, outdoor fashion, healing environment, design strategies

## Abstract

**Introduction:**

This study explores outdoor fashion as a medium for promoting psychological wellbeing, functional comfort, and sociocultural connection. As leisure, health, and identity converge, the research conceptualizes the components of healing environments, defines key healing design characteristics, and analyses their practical applications in outdoor fashion to support psychosocially responsive design strategies.

**Methods:**

A literature review established theoretical links between four types of healing environments (physical, functional, social, and psychological) and eight healing design characteristics (pleasantness, independence, accessibility, openness, safety, aesthetic quality, biophilic quality, and sociality). A case analysis of 805 products from six global outdoor brands (2021–2024) examined structural, pattern, material, and color elements. Coding reliability was verified through expert validation and Cohen’s Kappa analysis (*κ* = 0.74–0.76).

**Results:**

Outdoor fashion integrating healing design principles combined structural, pattern, material, and color elements that theoretically support psychological, physical, and social wellbeing. Ergonomic, semi-open, and adjustable structures enhanced comfort, independence, and safety; nature-inspired and geometric patterns strengthened biophilic connection and cultural identity; soft and reflective materials provided tactile comfort and perceived security; and color strategies influenced emotional stability, visibility, and aesthetic quality. Comfort, aesthetic quality, and psychological stability emerged as shared brand priorities, and the integrated use of multiple elements generated stronger healing effects than isolated features.

**Discussion:**

Findings indicate that outdoor fashion serves as a transdisciplinary platform that integrates psychological, functional, and sociocultural dimensions of care. Theoretical associations suggest that holistic combinations of design elements can enhance wellbeing by addressing sensory, emotional, and physical needs simultaneously. The proposed framework provides practical guidance for health-oriented design across commercial, community, and therapeutic contexts. However, these interpretations are theoretical rather than causal, and empirical user-based studies are required to validate the psychological and physiological associations identified.

## 1 Introduction

In contemporary society, evolving perceptions of leisure and health have driven the rapid expansion of outdoor fashion. No longer limited to activity-specific utility, outdoor fashion now merges functionality, style, and psychological wellbeing to address everyday lifestyle needs. Campaigns such as Arc’teryx’s *Outer Peace* illustrate how outdoor fashion is increasingly framed as a pathway to emotional and psychological healing (Frank, 2021). This trend aligns with a broader focus on healing-oriented design, evident in research on therapeutic medical clothing (Kam and Yoo, 2021; Zhao et al., 2024), wearable technologies and smart textiles for mental health (Ellouze and Damak, 2024), and fashion as a medium for cultural and emotional therapy (Clark, 2025). While these studies provide valuable theoretical insights and conceptual frameworks, systematic case-based analyses remain limited—particularly regarding how healing design elements are practically applied in outdoor fashion. Therefore, research is needed to examine how healing-oriented design strategies are implemented within contemporary outdoor fashion contexts to address psychological and physical wellbeing.

This study systematizes the components of healing environments and establish relationships among healing environments, healing design characteristics, and their intended effects. By analyzing how healing characteristics are expressed through structure, pattern, material, and color across leading outdoor fashion brands, this research clarifies design strategies that support psychological, physical, and social wellbeing in contemporary lifestyle contexts.

## 2 Literature review

### 2.1 Healing and healing environment

Healing is a multidimensional and integrative concept that encompasses physical, emotional, mental, and social recovery (DuBose et al., 2018; Huisman et al., 2012). Unlike clinical treatment, which typically targets the resolution of specific symptoms or diseases, healing is a holistic process aimed at restoring a person’s overall sense of wellbeing and inner balance. Healing in this study is defined as a perceived process of psychological, emotional, physical, and social restoration as experienced by the individual. This is distinct from clinical treatment, which targets the diagnosis and resolution of specific medical symptoms; the present work does not claim medical efficacy, but instead examines how design may theoretically support comfort, emotional stability, and social connection. The concept of healing differs among researchers across multiple disciplines. Mount et al. (2007) define healing as a process of achieving wholeness by integrating the physical, emotional, spiritual, and social dimensions of the individual, Egnew (2005) emphasizes the existential and psychological aspects of recovery, while Kabat-Zinn and Hanh (2009) highlights mindfulness as a cognitive-emotional practice that contributes to inner healing through presence and awareness. Despite these varying definitions and emphases, a common thread across these perspectives is that healing is not limited to biological recovery but is fundamentally linked to emotional regulation, psychological resilience, and social connectedness, dimensions that are increasingly important in contemporary design practice.

Healing design has been interpreted differently depending on disciplinary perspectives. Ulrich (1991) emphasized the role of natural elements in healthcare environments to promote recovery through stress reduction. Marcus and Barnes (1999) highlighted the importance of sensory and psychological responses in therapeutic spaces. Kam and Yoo (2021) demonstrated how visual elements such as symbolic patterns in patient clothing can enhance emotional stability and contribute to healing experiences. While approaches vary—from architectural environments to emotional clothing—the commonality lies in their focus on promoting physical, emotional, and psychological wellbeing through design, thereby supporting holistic health outcomes consistent with a biopsychosocial perspective.

A clear classification system is essential for the systematic analysis of healing design. According to previous studies, healing environments are closely linked to core concepts of healing and directly related to the goals of healing design. This study explored the correlation between healing characteristics and healing design expressions based on a classification system of healing environments. Based on a review of previous studies, healing environments can be categorized into physical, functional, social, and psychological environments.

The physical environment consists of natural and sensory elements—including natural light, colors, sounds, landscapes, and layouts—that directly influence users’ psychology and behavior through sensory stimulation (Dalke et al., 2006; Devlin and Arneill, 2003; Ulrich, 1991). The functional environment encompasses safety and convenience features through accident prevention design, ease of movement, and rational spatial arrangement, serving to promote physical and mental wellbeing and facilitate recovery (Gashoot, 2022; Tülbentçi and Arcan, 2021; Ulrich et al., 2008). The social environment provides spaces for interpersonal interaction and information exchange, enabling users to experience communication and develop psychological safety through social engagement (Tülbentçi and Arcan, 2021; Ulrich et al., 2008). The psychological environment is established through comfort, privacy, and security via aesthetically pleasing spaces with natural elements, strategic spatial organization, and appropriate lighting and color, contributing significantly to users’ psychological equilibrium and sustained wellbeing (Apriyanti et al., 2024; Tülbentçi and Arcan, 2021; Alkaisi et al., 2021; Mahmood and Tayib, 2019).

Collectively, these four environments provide the contextual foundation for understanding how design can influence wellbeing. However, to translate these environmental contexts into practical design applications, it is necessary to identify specific characteristics that convey healing experiences and to clarify how these characteristics are visually and materially expressed in design.

### 2.2 Healing design characteristics and expressions

Building upon the classification of Healing Environments discussed in Section 2.1, the characteristics of Healing Design were derived from previous studies that comprehensively examined the physical, functional, social, and psychological dimensions of healing. These characteristics represent the conceptual bridge through which environmental contexts are translated into tangible design strategies that support users’ wellbeing.

In this study, Healing Design Characteristics serve as the intermediary layer linking Healing Environments and the specific design elements that express them. Table 1 summarizes eight key characteristics (pleasantness, independence, accessibility, openness, safety, aesthetic quality, biophilic quality, and sociality) derived from the reviewed literature. Each characteristic can be implemented through distinct yet interrelated design elements, including structure, pattern, material, and color, which collectively shape users’ sensory and emotional experiences of healing.

**Table 1:** Healing design characteristics identified in previous studies.

Study	Healing design characteristics							
Ulrich et al. (1991)	✓			✓	✓		✓	
Devlin and Arneill (2003)	✓		✓		✓	✓		
Dijkstra et al. (2006)	✓			✓		✓	✓	
Ulrich et al. (2008)	✓	✓			✓	✓		
Huisman et al. (2012)			✓		✓	✓	✓	
Browning et al. (2014)	✓		✓	✓	✓	✓	✓	✓
Gesler (1992)	✓		✓		✓		✓	✓
Aristizabal et al. (2021)	✓	✓				✓	✓	
This study	Pleasantness	Independence	Accessibility	Openness	Safety	Aesthetics	Biophilic quality	Sociality

Pleasantness is realized through the promotion of comfort in various aspects. Key design expressions include ergonomic design with appropriate physical properties for physical comfort (Ulrich et al., 2008; Bai et al., 2024), control of environmental factors such as temperature, humidity, and air quality (Ulrich et al., 2008), selection of a calming color palette for visual comfort (Kellert et al., 2008), and spatial organization that reduces cognitive load and provides psychological tranquility (Ulrich et ai., 1991). In addition, the integration of natural elements such as plants, water, and natural light contributes to positive emotional responses and the promotion of wellbeing (Ulrich et al., 1991; Browning et al., 2014). Environments that embody such comfort can effectively contribute to reducing stress, increasing resilience, and enhancing psychological wellbeing, thereby supporting overall life satisfaction.

Independence reflects the user’s sense of control and autonomy within the space. Studies by Ulrich et al. (2008) and Dijkstra et al. (2006) show that being able to control environmental elements such as lighting and temperature, or providing a personal space, can increase a user’s sense of agency, which can have positive psychological consequences. Design expressions include single rooms, modular layouts, and customized environments support autonomy and reduce stress by allowing individuals to control their surroundings. Gesler (1992) emphasized the importance of providing space options for a variety of preferences and activities, and Evans and McCoy (1998) suggested ways to design flexible environments that can be modified by users.

Accessibility refers to the ease with which users can navigate, understand, and physically access a space, regardless of their physical or cognitive abilities. Such an environment promotes user confidence, reduces anxiety, and enhances the user’s ability to make decisions and act on their own. Design expressions include emphasizing the importance of barrier-free design, clear circulation patterns, and wayfinding aids (Devlin and Arneill, 2003; Dijkstra et al., 2006), implementing universal design principles throughout the space (Browning et al., 2014), incorporating legible signage with appropriate contrast and size (Rodrigues et al., 2019), and providing appropriate furniture heights and designs for different users (Blackler et al., 2018).

Openness is defined by spatial transparency, unobstructed views, and visual connections to a broader environment. As posited by Ulrich et al. (1991), exposure to unspoiled natural environments has been demonstrated to attenuate psychological distress and sensations of peril, thereby engendering salutary emotional responses. As Browning et al. (2014) elucidate, openness fosters psychological stability by balancing a broad perspective with a sense of safety through protective spaces. Design elements expressing openness include generous spatial proportions (Yan et al., 2024), transparent or translucent partitions that promote visual connections (Ulrich, 1984), forming sightlines that connect different functional areas (Gesler et al., 2004), and minimizing unnecessary walls and barriers (Conradson, 2005).

Safety is a concept that encompasses two aspects: physical protection and psychological security. Physical safety focuses on preventing injury and danger, while psychological safety emphasizes the comfort and stability that users feel within an environment. Ulrich et al. (2008) found that environmental factors such as ergonomic layout, slip-resistant materials, and clear spatial organization contribute to enhancing safety. Intuitive spatial layouts reduce users’ anxiety and enhance their confidence. Design expressions that implement safety include the use of rounded edges on furniture and architectural elements, the removal of tripping hazards, and visually clear access points that consider security and accessibility (Gesler, 1992).

Aesthetic Quality refers to the visual and sensory harmony of an environment, including materials, forms, colors, and symbolic elements that resonate with users. According to Devlin and Arneill (2003), aesthetics are important in creating an emotional environment, and Browning et al. (2014) demonstrated that aesthetic patterns such as biomorphism, complexity, and order are associated with human perceptual preferences. A study by Aristizabal et al. (2021) showed that visually appealing environments improve user satisfaction and cognitive performance. Methods for implementing Aesthetic Quality include incorporating artistic elements within a space (Foster et al., 2025), applying a balanced color theory (Ghamari and Amor, 2016), designing spaces with proportion and rhythm (Kellert et al., 2008), incorporating visual elements that reflect cultural preferences (Park and Park, 2013), using materials with tactile qualities, and implementing a consistent design language (Browning et al., 2014).

Biophilic quality is a concept that integrates natural elements or elements that evoke nature into design. Connection with nature enhances stress recovery, mood improvement, and overall wellbeing. Methods of expressing nature-friendly design include providing natural views through windows and utilizing natural lighting (Kellert and Calabrese, 2015; Ulrich, 1984), indoor vegetation (potted plants, living walls, indoor gardens) and water features (Browning et al., 2014), using natural materials such as wood, stone, and natural fibers (Augustin and Fell, 2015), and applying organic patterns and forms to architectural and interior elements (Kellert et al., 2008).

Sociality promotes positive social interaction and support, strengthening emotional bonds and enhancing healing experiences (Huisman et al., 2012; Ulrich et al., 2008). Design expressions include creating various spaces that support interaction (Gesler, 1992) and constructing traffic patterns that create opportunities for spontaneous interaction (Browning et al., 2014).

**Figure 1:**
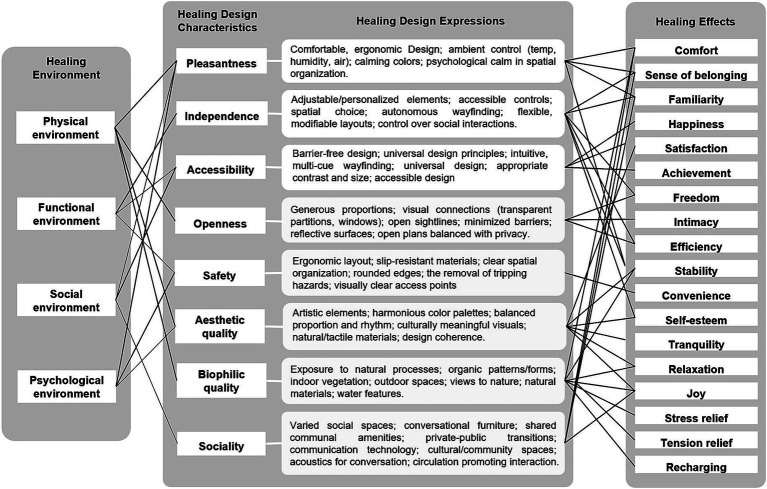
Relationship between healing design characteristics, design expression, and healing effects.

Overall, the conceptual hierarchy among these dimensions can be summarized as follows: Healing Environments establish the contextual foundation that gives rise to specific Healing Design Characteristics, which are subsequently manifested through tangible Healing Design Expressions—including structure, pattern, material, and color. These expressions collectively generate theoretical Healing Effects that support users’ psychological, physical, and social wellbeing. Figure 1 illustrates this relationship, showing how Healing Environments underpin Healing Design Characteristics, which are expressed through design expressions leading to corresponding Healing Effects.

### 2.3 Outdoor fashion and healing

The global COVID-19 pandemic has heightened public interest in health and wellbeing, leading to increased participation in outdoor activities such as hiking, camping, and cycling (Singh and Singh, 2025). Consequently, the outdoor apparel market has shown consistent worldwide growth. According to Global Market Insight (2024), the global outdoor apparel and accessories market was valued at approximately USD 37.1 billion in 2024 and is projected to continue expanding due to the increasing popularity of recreational activities and health-oriented lifestyles. Specifically, the domain of outdoor fashion is witnessing a shift in focus from its historical emphasis on hiking to encompass a broader array of leisure activities, including trekking, cycling, and camping. This evolution is accompanied by a significant expansion in the range of products designed specifically for these activities. The outdoor fashion market is undergoing a rapid transformation, characterized by the convergence of leisure activities and fashion (Penouty, 2024).

Numerous studies have demonstrated the healing benefits of outdoor activities, showing that exposure to natural environments promotes stress reduction, mood enhancement, and physiological recovery (Ulrich, 1984; Kaplan and Kaplan, 1989; Hartig et al., 2014). The experience of nature has been demonstrated to be closely associated with increased happiness, improved social participation, enhanced ability to manage life challenges, and reduced mental distress (Bratman et al., 2015; Mitten et al., 2018; Hunter et al., 2019; Ma et al., 2024). Based on these research findings, the outdoor wear industry has adopted healing as a key concept and is implementing marketing strategies focused on stress and anxiety relief, as well as overall physical and mental health improvement (Kim, 2012).

Outdoor clothing has been shown to offer both functionality and healing effects through evidence-based design grounded in ergonomics and sports science. According to research in ergonomic design, garment fit and structural properties significantly influence physical comfort and movement efficiency during outdoor activities (Bai et al., 2024). From sports sciences perspective, ensuring dryness and maintaining optimal body temperature are of importance for the comfort of the wearer. Materials that possess breathability, moisture-wicking properties, and insulating capabilities assist wearers in effectively sustaining ideal body temperature (Li, 2005). Furthermore, multifunctional designs that incorporate moisture management, windproof barriers, and waterproof membranes help maintain thermal regulation and dryness during various outdoor activities, supporting both physical comfort and psychological wellbeing (Zhu et al., 2025). These functional characteristics not only enhance practical performance but also facilitate the wearer’s connection to nature, allowing for a more stable and comfortable experience in outdoor environments (Cartner-Morley, 2024).

Recent industry developments illustrate the integration of healing-oriented design in outdoor fashion products. FILA collaborated with BBC Earth on a project called “From Nature to Nature.” The objective of the project is to attract urban residents to nature, thereby reducing stress and fostering emotional stability. The clothing is designed with windproof, waterproof, and breathable properties to facilitate a harmonious integration with the natural environment (Qu, 2023). Blackyak’s “Cool Anywhere Vacation Collection” has been developed to address the contemporary inclination toward healing and relaxation. This collection utilizes functional materials with moisture-wicking and cooling effects, combining graphic design elements with natural motifs to evoke the ambience of a resort setting. The collection aims to enable wearers to experience a sense of wellbeing and vitality, even in high-temperature conditions (Kwak, 2020).

Consequently, the domain of outdoor fashion has evolved beyond the category of simple functional clothing to become a medium for emotional healing that promotes psychological recovery and stress relief through exposure to natural environments. The functional characteristics of these garments, including waterproofing, wind proofing, and breathability, offer protection and comfort to wearers in various environmental conditions. These characteristics enhance immersion in natural surroundings and facilitate the experience of physical and mental relaxation and freedom. Furthermore, the integration of contemporary trends with natural elements in outdoor fashion facilitates a harmonious connection between urban life and nature, thereby integrating aesthetics and functionality to further enhance emotional healing effects. This shift indicates an evolution in outdoor fashion, aligning with the contemporary societal trend toward wellbeing.

## 3 Methods

### 3.1 Research subjects and scope

The dataset for this study consisted of official product images from six leading outdoor fashion brands—Patagonia, Arc’teryx, Marmot, Columbia Sportswear, The North Face, and Cotopaxi—selected for their global market influence, product diversity, and strong brand identity in outdoor apparel (Nelson, 2024). A total of 884 product images published on brand websites between 2021 and 2024 were collected. After excluding those with unclear visuals or incomplete product information, 805 products with clearly identifiable design features remained for final analysis. The 2021–2024 period represents a phase of post-pandemic growth in outdoor apparel and a rising focus on healing-oriented design. All analyzed images were official product photographs retrieved from brand websites to ensure authenticity and consistency. Only standardized front, back, and detailed product shots were used; editorial and lifestyle campaign images were excluded to avoid bias from contextual styling or photographic effects. This ensured consistency and minimized the influence of external factors such as lighting, models, or backgrounds. Each image was treated as a single analytical unit representing an individual product. Product descriptions and specifications provided on brand websites verified the item’s identity and accuracy. All images were stored and coded in a unified dataset categorized by brand and product type (e.g., jacket, vest, pants, accessories). This transparent sampling process ensured that the dataset provided a consistent and representative foundation for the coding and reliability analyses described in Section 3.2. Although the selected brands include both premium and mid-range categories, future research should expand to broader price ranges to avoid sampling bias.

### 3.2 Coding procedure and reliability assurance

The analysis was conducted through a systematic validation process involving five stages (see Sections 3.2.1–3.2.5) to ensure reliability and objectivity. This multi-stage approach aligns with qualitative validation principles proposed by Lincoln and Guba (1985) and Creswell and Poth (2018).

#### 3.2.1 Development of coding framework

**Figure 2:**
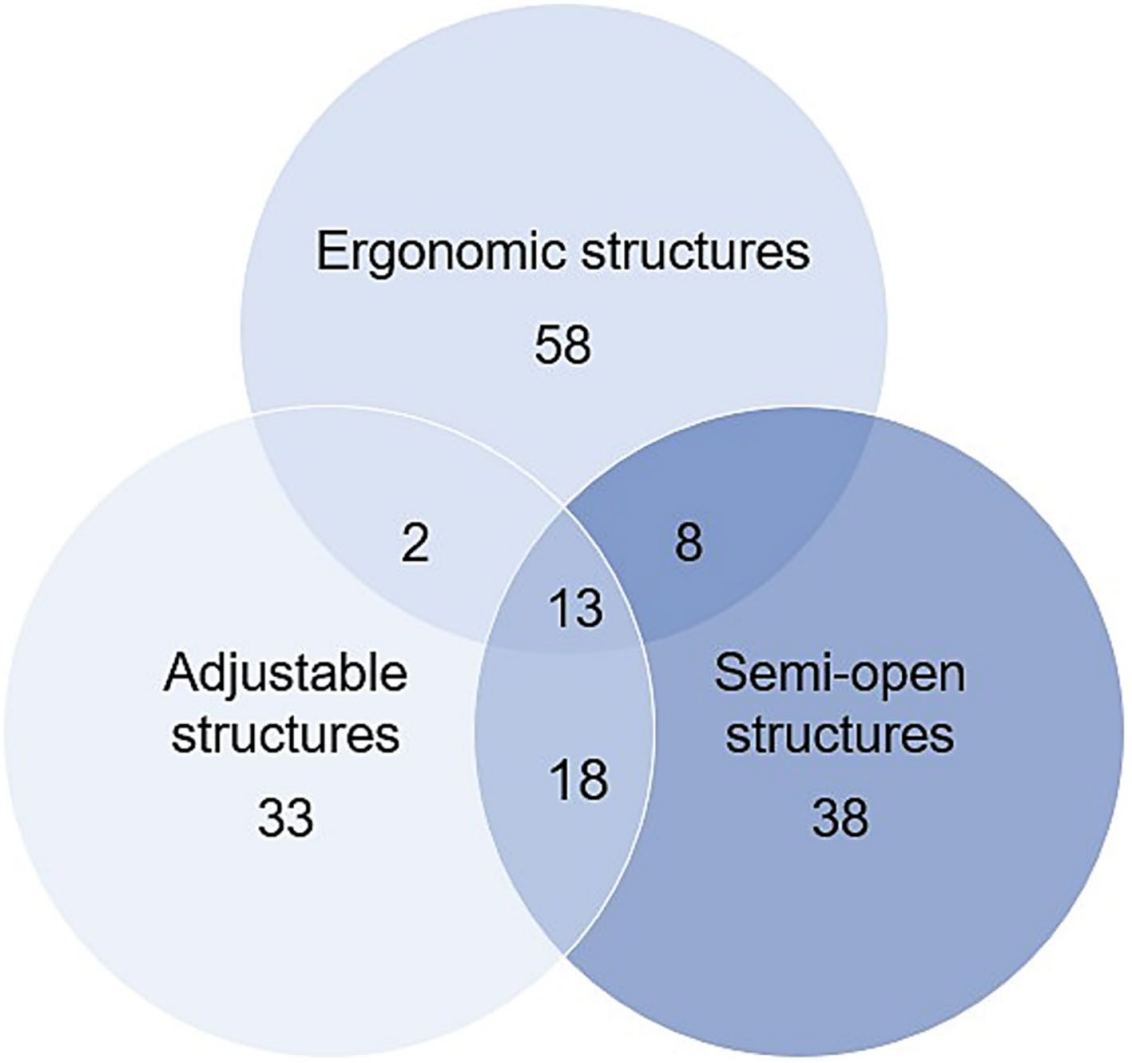
Healing design expressions through structures (*n* = 170).

A detailed codebook was developed through iterative discussions among the research team. The codebook provided: (1) operational definitions for eight Healing Design Characteristics (pleasantness, independence, accessibility, openness, safety, aesthetic quality, biophilic quality, and sociality) derived from the theoretical framework presented in the literature review (Figure 1); (2) classification criteria for four Design Elements (structure, pattern, material, and color) with visual examples from the preliminary product review; and (3) standardized coding rules to guide consistent decisions in ambiguous cases.

#### 3.2.2 Coder selection and training

Two researchers with expertise in fashion design and healing-oriented design served as independent coders. Coder 1 (Ph.D. in Fashion Design, 8 years of research experience in fashion and healing design) and Coder 2 (M.A. in Fashion Design, expertise in fashion design analysis) had no financial or professional ties to the brands studied. Both completed training through pilot coding of 50 sample products to refine definitions and ensure a shared understanding of classification criteria.

#### 3.2.3 Independent coding and reliability

The two coders independently analyzed all 805 product images using the established codebook. For each product, they identified (1) which Design Elements were present and (2) which Healing Design Characteristics were expressed through these elements. Inter-coder reliability was assessed using Cohen’s Kappa coefficient. Both Design Elements classification (*κ* = 0.76) and Healing Design Characteristics identification (*κ* = 0.74) demonstrated substantial agreement (0.61 ≤ *κ* ≤ 0.80), according to Landis and Koch (1977).

#### 3.2.4 Discrepancy resolution and expert validation

Coding discrepancies were reviewed through a structured consensus process. The coders first re-evaluated cases using the codebook and theoretical framework. Unresolved cases were referred to a six-member expert panel consisting of one fashion design professor, two outdoor industry professionals, and three researchers in fashion design (average experience = 9 years). Consensus was reached when at least five of six experts agreed. This procedure ensured methodological rigor and strengthened the content validity of the coding results.

#### 3.2.5 Triangulation of healing effect associations

Associations among Healing Design Characteristics, Outdoor Healing Design Elements, and Healing Effects were validated through methodological triangulation using three complementary evidence sources:

1. The coded results of 805 products, which provided descriptive evidence of how Healing Design Characteristics were visually expressed through four Design Elements—structure, pattern, material, and color. These results formed the empirical basis for identifying recurring patterns and co-occurrences among Design Elements and healing characteristics.
2. The expert validation process, in which two coders and six external specialists reviewed and confirmed the plausibility, clarity, and theoretical consistency of the coded results. Discrepancies were resolved through structured consensus discussions, and agreement by at least five of six experts was considered valid.
3. The theoretical framework established in Section 2, which grounded the interpretation of associations within existing research in environmental psychology, biophilic design, and healing design theory. This framework ensured that the inferred relationships between Design Elements and Healing Effects aligned with established theoretical constructs.

#### 3.2.6 Ethical considerations

Based on the nature of the data and the absence of human participants or personal information, ethical approval was not required for this study. The analysis was conducted exclusively on publicly available product images retrieved from official brand websites.

## 4 Outdoor fashion design from a healing perspective

### 4.1 Analysis of healing expressions in fashion design elements

A total of 805 products were analyzed according to four fashion design elements: structures, patterns, materials, and colors, to examine how healing-oriented expressions appear across outdoor apparel. When multiple design elements coexisted within a single product, they were systematically coded as individual elements and subsequently aggregated to identify their distinctive characteristics. The descriptive analysis identified 170 cases of healing design expression through structures, 178 cases through patterns, 107 cases through materials, and 337 cases through colors. Percentages shown in Table 2 represent the proportion of products within each brand that exhibited the corresponding healing design expression, calculated based on each brand’s total sample size (*n* = 134 or 135). For example, 21.6% of Arc’teryx products (29 out of 134) incorporated ergonomic structures, while 23.1% (31 out of 134) featured semi-open ventilation structures. The “Total (%)” column indicates the overall proportion of each design element category across the entire dataset (*n* = 805). Because individual products could contain multiple design elements and thus contribute to more than on categories, column percentages may exceed 100%. These values describe descriptive tendencies only and do not represent results of statistical testing.

**Table 2:** Healing design expressions by brand and design element (%).

Design element	Category	Patagonia	Arc’teryx	Marmot	Columbia sports	The north face	Cotopaxi	Total (*n* = 805)
Structures	Ergonomic structures	0.7	21.6	8.2	6.7	19.3	3.7	10.1
	Semi-open structures	1.5	23.1	18.7	3.7	9.6	0.7	9.6
	Adjustable structures	2.2	18.7	9.7	6.7	11.9	0.0	8.2
Patterns	Nature-inspired patterns	16.4	1.5	6.7	11.2	28.1	9.7	12.3
	Geometric patterns	20.1	0.7	11.2	11.9	11.1	11.9	11.2
Materials	Soft materials	23.1	6.0	6.0	14.9	11.9	11.9	12.3
	Reflective materials	0.0	2.2	0.0	1.5	2.2	0.0	1.0
Colors	Nature-inspired colors	17.9	17.2	4.5	5.2	24.4	10.4	13.3
	High-contrast colors	17.2	4.5	20.9	17.9	31.1	41.8	22.2
	Soft colors	9.0	6.0	0.7	11.2	26.7	6.7	10.1
	Fluorescent colors	5.2	11.2	3.7	3.7	12.6	0.7	6.2

Percentages in each brand column are calculated within the brand’s sample (*n* = 134 for Patagonia, Arc’teryx, Marmot, Columbia, and Cotopaxi; *n* = 135 for The North Face). “Total (%)” represents the overall proportion of each design element category across all 805 products included in the analysis.

#### 4.1.1 Structure

As shown in Figure 2, healing design expressions through structures were classified into ergonomic structures (*n* = 81), semi-open structures (*n* = 77), and adjustable structures (*n* = 66). Analysis revealed that these structural elements often coexisted within single products, with 21 cases showing overlap between ergonomic and semi-open structures, 31 cases between semi-open and adjustable structures, and 15 cases between adjustable and ergonomic structures. A total of 13 cases incorporated all three. These descriptive results indicated that outdoor garments frequently integrate multiple structure types rather than applying them in isolation. Brand-specific frequencies and proportions are detailed in Table 1. The classification and coding of structural features were based on the Healing Design Framework established through the literature review (Section 2.2), which linked specific design expressions to Healing Environment types and Healing Design Characteristics.

Ergonomic structures are expressed through designs that consider the curves of the human body and structural arrangements that optimize mobility. Three-dimensional cutting techniques were employed to minimize fabric excess, friction, and movement restriction. These features were coded as expressions of the healing design characteristics pleasantness, accessibility, and safety, as defined in the analytical framework (Bai et al., 2024; Ulrich et al., 2008; Huisman et al., 2012). Semi-open structures incorporated ventilation features such as underarm zippers and breathable side panels to facilitate air circulation during activity. These design expressions are associated with the healing design characteristics of pleasantness, accessibility, and openness (Evans and McCoy, 1998; Browning et al., 2014; Kellert and Calabrese, 2015). The adjustable structures provide users with the ability to achieve personalized comfort, resulting in a stable and adaptable wearing experience. Adjustable structures included modifiable elements such as hoods, cuffs, and waistbands, enabling personalized fit customization. This structural approach aligns with the healing design characteristics of pleasantness, independence, accessibility, and safety (Evans and McCoy, 1998; Huisman et al., 2012; DuBose et al., 2018; Devlin and Arneill, 2003).

#### 4.1.2 Patterns

**Figure 3:**
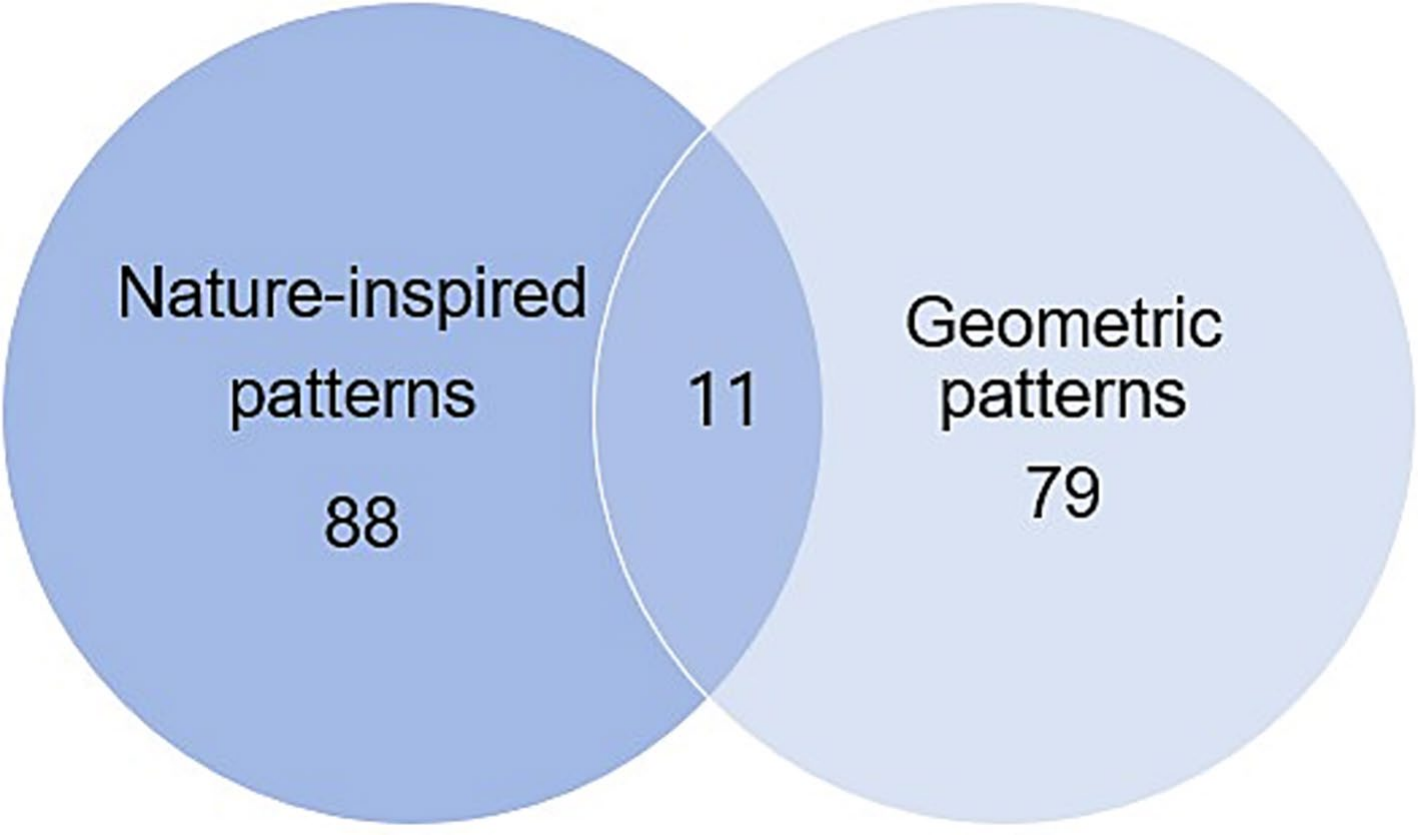
Healing design expressions through patterns (*n* = 178).

Healing design expressed through patterns can be broadly classified into two categories: nature-inspired patterns and geometric patterns. Figure 3 illustrated the overlapping relationships between pattern types, showing how healing design is expressed through nature-inspired and geometric motifs. The descriptive analysis identified 99 cases of nature-inspired patterns and 90 cases of geometric patterns, with 11 cases where both types coexisted. These results suggest that outdoor fashion tends to combine natural and geometric visual languages, reflecting a balanced approach rather than reliance on a single motif system.

Nature-inspired patterns are expressed through realistic or abstract depictions of natural landscapes, flora, and geographical features such as mountains or waves. They appear as both realistic and abstract expressions, characterized by organic rhythm, flowing composition, and color harmony derived from nature. Within the framework of healing design, these motifs are closely related to the healing design characteristics of pleasantness, biophilic quality, and aesthetic quality, as they visually evoke comfort, familiarity, and connection with natural environments (Aristizabal et al., 2021; Ghamari and Amor, 2016; Browning et al., 2014). Geometric patterns use structured shapes, symmetry, and rhythmic repetition to express visual order and cultural meaning. These patterns often reinterpret forms derived from nature or ethnic motifs to convey intimacy and visual harmony. From a healing design perspective, these motifs are closely related to the healing design characteristics of aesthetic quality, biophilic quality, and sociality (Kellert and Calabrese, 2015; Browning et al., 2014; Aristizabal et al., 2021).

#### 4.1.3 Materials

Healing design expressions through materials were classified into two primary types: soft materials and reflective materials. A total of 107 cases were identified across the dataset. These findings indicate that outdoor fashion commonly applies material properties that promote tactile comfort and perceptual stimulation, supporting both physical and emotional aspects of healing design.

Soft materials—such as fleece, padded textiles, and lightweight knits—were widely used to create warmth and tactile comfort. Soft materials deliver relaxation and stability through a gentle tactile experience. These materials align with the healing design characteristics of pleasantness (Browning et al., 2014; Pasqualotto et al., 2020). Reflective materials—such as metallic coatings, glossy laminations, and high-visibility surfaces—served dual purposes, addressing both functional performance and expressive aesthetics. Within the framework of healing design, these reflective elements were applied to clothing components such as hats, structural joints, and seam lines. They enhance decorative and aesthetic appeal through dynamic light effects generated by movement and simultaneously provide a sense of safety and protection in outdoor environments, which may contribute to users’ self-esteem (Browning et al., 2014; Kim and Song, 2021).

In summary, material-based healing design elements bridge tactile relaxation and visual stimulation. Soft materials evoke pleasantness and stability through sensory comfort, while reflective materials express stimulation and safety through perceptual engagement.

#### 4.1.4 Colors

Healing design expressed through color can be classified into four categories: nature-inspired colors, high-contrast colors, soft colors, and fluorescent colors. These findings indicate that outdoor fashion commonly employs color strategies that evoke emotional relaxation, perceptual stimulation, and environmental harmony.

Nature-inspired colors, including greens, browns, and sky-like blues, reflect tones found in natural environments. Within the framework of healing design, these colors correspond to the healing design characteristics of pleasantness, biophilic quality and aesthetic quality (Ulrich et al., 2008). They enhance users’ connection with nature, provide visual balance, and promote psychological restoration through familiarity and ecological resonance (Kellert and Calabrese, 2015). High-contrast colors—such as vivid complementary pairings or strong light–dark combinations—create striking visual stimulation and accentuate structural details. In the context of healing design, high-contrast colors evoke positive emotions through visual emphasis and convey aesthetic sensibility through color combinations. These vivid color combinations provide wearers with joy and comfort while enhancing visibility during outdoor activities, thereby improving safety (Ghamari and Amor, 2016; Rodrigues et al., 2019). Soft colors—such as pastels, beige, and muted tones—convey calmness and emotional warmth. These hues are often employed to create a natural and visually balanced composition, offering the wearer a warm, peaceful, and harmonious sensory experience. They correspond to the healing design characteristics pleasantness, and aesthetic quality, which reduce visual tension and thereby evoke healing effects such as comfort and approachability (Jonauskaite and Mohr, 2025; Ou et al., 2004). Fluorescent colors, such as neon yellow, orange, or green, express vitality and energetic visibility. Within healing design, they are associated with stimulation and safety, reinforcing users’ sense of liveliness and protection—particularly in dynamic or low-light environments. Their luminous intensity elicits alertness and sensory awareness, encouraging active engagement with surroundings (Kim and Song, 2021). In addition, these fluorescent hues are often used by outdoor fashion brands to express their distinct visual identity and energetic brand style, functioning as a symbolic element that differentiates their products within the market.

### 4.2 The relationship between healing design characteristics, outdoor healing design elements, and healing effects

**Figure 4:**
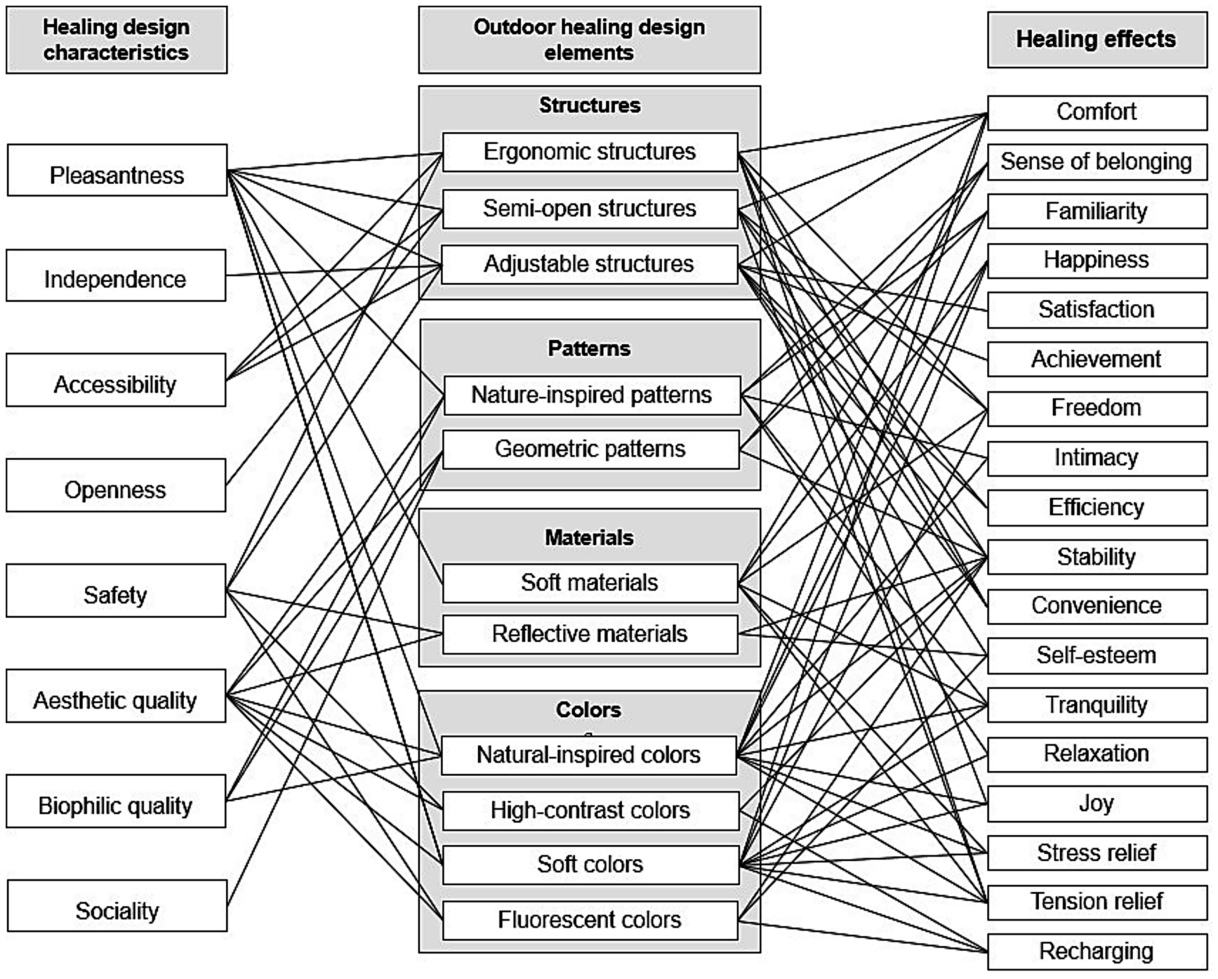
The relationship between healing design characteristics, outdoor healing design elements, and healing effects.

This section interprets how the Healing Design Characteristics identified in Section 4.1 theoretically correspond to Healing Effects established in environmental psychology and healing design literature. The interpretation is grounded in three evidence sources: (1) the coded results of 805 products, (2) the expert validation process described in Section 3.2.5, and (3) the theoretical framework established in Section 2. The study did not measure actual user experiences, psychological states, or physiological responses. Therefore, all healing effects discussed below represent theoretically grounded possibilities rather than empirically verified outcomes for these specific products. Figure 4 visualizes the theoretically grounded associations among Healing Design Characteristics, Outdoor Healing Design Elements, and their potential Healing Effects derived from the case analysis. Based on established design theory, these elements are conceptually interconnected and may collectively support restorative experiences that contribute to users’ holistic wellbeing—though empirical validation with actual users is needed to confirm these theoretical relationships.

As shown in Table 2, ergonomic structures were the most prevalent among structural expressions, accounting for approximately 10.1% of all products (81 cases in total). Semi-open structures (9.6%) and adjustable structures (8.2%) were also frequent, indicating that structural elements in outdoor fashion commonly integrate multiple functional features that support mobility and comfort. According to ergonomic design research (Kim and Song, 2021; Huisman et al., 2012), such structural features in apparel and built environments have been linked to improved freedom of movement and reduced physical strain. Drawing from this body of research, three-dimensional tailoring in outdoor garments may theoretically support the body’s natural motion and prevent restriction, potentially promoting stability, joy, and tension relief. Semi-open structures, such as ventilation ports and underarm zippers positioned on the back or sides, were also prevalent. These features are designed to facilitate airflow and mitigate heat buildup during activity. Healing environment studies (Browning et al., 2014) suggest that enhanced thermal comfort can be associated with efficiency and psychological ease in built environments—indicating that similar ventilation strategies may provide freedom, convenience, and tension relief in outdoor contexts. Adjustable components—including cords, elastic bands, and Velcro straps—allow users to control fit and tightness according to individual needs. Research on environmental control in healthcare settings (Ulrich et al., 2008; DuBose et al., 2018) indicates that user autonomy over environmental features can enhance satisfaction, self-esteem, and psychological stability. By extension, adjustable apparel may produce similar benefits by fostering a sense of autonomy and personalization. Collectively, these structural strategies are designed to enhance physical ease and psychological empowerment.

Pattern elements are theoretically positioned to evoke familiarity and a sense of belonging. Nature-inspired patterns—such as motifs depicting leaves, mountains, or clouds—account for approximately 12.3% of all products (99 cases). Reflecting organic forms, these designs align with biophilic design principles (Kellert and Calabrese, 2015) that emphasize psychological restoration and connection with nature. These patterns may facilitate reconnection with natural environments and support intimacy, tranquility, and relaxation. Geometric patterns (11.2%, 90 cases) emphasize order and stability. Visual perception studies (Aristizabal et al., 2021) indicate that structured motifs can provide psychological grounding and balance in built environments. When combined with folk or regional references, these patterns may reinforce cultural attachment and expressive identity (Gesler, 1992), potentially contributing to belonging, stability, and joy.

Material elements are designed to influence tactile and perceptual experiences related to healing effects. Soft materials—such as fleece, padded textiles, and stretch fabrics—account for approximately 12.3% of all products (99 cases). These materials provide tactile softness and body responsiveness. Tactile perception research (Pasqualotto et al., 2020) suggests that soft textures can be associated with comfort and relaxation. Within healing design frameworks (Browning et al., 2014), gentle tactile experiences have been linked to stress relief and emotional ease. Reflective materials—such as glossy laminations and metallic coatings—account for approximately 1.0% of all products (8 cases). These reflective surfaces are applied to enhance visibility and convey a sense of protection, symbolically reinforcing safety and alertness in outdoor environments. Research on high-visibility apparel (Kim and Song, 2021) indicates that enhanced visibility can support safety awareness and self-esteem in outdoor environments.

Color elements are theoretically associated with diverse healing effects. Color psychology research (Ulrich et al., 2008; Jonauskaite and Mohr, 2025) suggests that nature-inspired and soft colors can be linked to relaxation, tranquility, and comfort by reducing visual tension and evoking gentle sensory harmony. In contrast, studies on high-visibility design (Ghamari and Amor, 2016; Kim and Song, 2021) indicate that high-contrast and fluorescent colors have been associated with enhanced vitality, alertness, and safety awareness during outdoor activities.

As summarized in Figure 4 and Table 2, products that were found to incorporated multiple design elements—especially combinations of ergonomic structures, soft materials, and harmonized color palettes-were more frequently coded with multiple healing effects (e.g., comfort, relaxation, stability, and joy) than those emphasizing a single element. This descriptive trend, validated through expert consensus review (Section 3.2.5), suggests that the coexistence of complementary design elements may generate synergistic healing effects. For example, comfort and relaxation appeared most frequently in products combining ergonomic structures with soft, tactile materials; stability and happiness in those featuring reflective surfaces and high-contrast colors; and tranquility in products integrating nature-inspired patterns, soft materials, and muted color tones.

However, these observed relationships represent interpretive linkages rather than experimentally verified causal effects. The findings indicate, rather than confirm, that outdoor fashion tends to provide stronger healing experiences when multiple design elements interact holistically to address physical comfort, emotional relaxation, and psychological stability together. Future research involving user testing and physiological or psychological measurement is recommended to empirically verify these multidimensional healing associations.

### 4.3 Brand-specific strategies and practical implications of healing design in outdoor fashion

#### 4.3.1 Comparative brand strategies

This section analyses the Healing Design Characteristics implemented by six outdoor fashion brands—Patagonia, Arc’teryx, Marmot, Columbia Sportswear, The North Face, and Cotopaxi—based on the theoretical framework established in Section 2. Table 3 illustrates each brand’s distinct approach to expressing healing design characteristics through design elements.

**Table 3:** Healing design expressions in outdoor fashion brands.

Characteristics of healing design	Outdoor fashion brand healing design expression methods					
	Patagonia	Arc’teryx	Marmot	Columbia	The north face	Cotopaxi
Pleasantness	Patterns featuring natural landscapes Fleece material Nature-inspired colors Soft colors	Ergonomic 3D cutting Semi-open ventilation structure Adjustable hood structure Nature-inspired colors Soft colors	Reinforced joint area structure Semi-open ventilation structure Adjustable waistband cuffs, and hood.	Nature-inspired patterns Fleece material Stretchy fabric Soft colors	Body-contouring structure Reinforced joint areas Nature-inspired patterns Nature-inspired colors Soft colors	Nature-inspired cartoon patterns Fleece material Stretchable fabric Nature-inspired colors
Independence		Adjustable structure	Adjustable structure		Adjustable size and elastic structure	
Accessibility		Ergonomic structure Semi-open structure Adjustable structure.	Ergonomic structure Semi-open structure, Adjustable structure.	–	Ergonomic structure Semi-open structure Adjustable structure.	–
Openness		Semi-open ventilation structure	Semi-open ventilation structure	–	Semi-open ventilation structure	–
Safety	Fluorescent colors	Ergonomic structure Adjustable structure Reflective material Fluorescent colors	Ergonomic structure Adjustable structure High contrast colors	Reflective material High contrast colors	Ergonomic structure Reflective material High contrast colors Fluorescent colors	High contrast colors
Aesthetic quality	Nature-inspired patterns Geometric patterns Nature-inspired colors Soft blue and pink colors	Reflective material Nature-inspired colors, fluorescent colors	Geometric patterns	Nature-inspired patterns Geometric patterns Reflective material Nature-inspired colors Soft colors	Nature-inspired patterns Geometric patterns Reflective material Nature-inspired colors High contrast colors Soft colors Fluorescent colors	Nature-inspired patterns Nature-inspired colors High contrast colors
Biophilic quality	Nature-inspired patterns Geometric patterns Nature-inspired colors	Nature-inspired colors	Geometric patterns	Nature-inspired patterns Geometric patterns	Nature-inspired Patterns Geometric patterns Nature-inspired colors	Nature-inspired patterns Nature-inspired colors
Sociality	Ethnic geometric pattern			Ethnic geometric pattern	Geometric patterns created through collaboration with artists	

Patagonia expressed pleasantness through natural landscape patterns, fleece materials, nature-inspired and soft colors, while enhancing safety through fluorescent accents. It also incorporated aesthetic quality, biophilic quality, and sociality via geometric and folk-inspired motifs, though independence, accessibility, and openness were less emphasized.

Arc’teryx prioritized pleasantness, independence, accessibility, and openness through ergonomic, adjustable, and semi-open ventilation structures. Safety was strengthened through functional detailing, reflective surfaces, and fluorescent color usage. Aesthetic and biophilic qualities were expressed through nature-inspired palettes, but sociality was limited.

Marmot emphasized pleasantness and accessibility using reinforced joints, semi-open ventilation, and adjustable details. Geometric patterns and high-contrast colors highlighted safety and aesthetic quality, though sociality remained secondary.

Columbia Sportswear actively utilized nature-inspired patterns, soft colors, and tactile materials such as fleece and stretch fabrics to evoke pleasantness, Safety and aesthetic quality were expressed through reflective materials and high-contrast colors. While accessibility and independence were less pronounced.

The North Face reinforced pleasantness via body-fitting three-dimensional structure and joint reinforcement, and independence through adjustable elastic components. Safety was expressed through fluorescent hue and reflective detailing while biophilic quality and sociality were achieved through nature-inspired and artist-collaboration patterns.

Cotopaxi applied cartoon-like, nature-inspired patterns and stretchy fleece material to enhance pleasantness, and emphasized safety and aesthetic quality through high-contrast color combinations. However, independence, accessibility, openness, and sociality were underrepresented.

In conclusion, each brand strategically selected and utilized different healing design characteristics in accordance with their own design philosophy and brand identity. Notably, all brands emphasized comfort and aesthetic quality to varying degrees, suggesting these are core values in healing-oriented outdoor fashion design. The variation in approach—from Arc’teryx’s structural-functional focus to Patagonia’s material-pattern emphasis to The North Face’s comprehensive integration—demonstrates multiple pathways for implementing healing design principles in commercial outdoor fashion contexts.

#### 4.3.2 Practical design guidelines

The analysis of brand-specific strategies reveals several key principles for integrating healing design into outdoor fashion products. First, the most effective healing design expressions resulted from combining multiple complementary design elements rather than relying on isolated features. The North Face’s integration of ergonomic structures, nature-inspired patterns, soft materials, and varied colors aligned with six to seven Healing Design Characteristics simultaneously, while single-element approaches addressed fewer dimensions. This suggests that designers should strategically combine at least two to three elements—such as ergonomic structure, soft material, and nature-inspired color—to create synergistic healing effects.

Design priorities should be aligned with intended use context to maximize effectiveness. For technical outdoor activities requiring high performance and safety, brands like Arc’teryx demonstrate the value of prioritizing independence, accessibility, and safety through adjustable structures and high-visibility elements. In contrast, for urban outdoor and lifestyle use, the Patagonia model shows how emphasizing pleasantness and biophilic connection through nature-inspired patterns, soft materials, and harmonious colors can better serve user needs. For versatile, multi-activity products, The North Face’s approach of balancing functional and aesthetic dimensions provides a useful reference.

Material-color synergy emerged as a critical factor in shaping emotional impact. The analysis revealed that soft materials paired with nature-inspired or soft colors consistently aligned with pleasantness and relaxation, while reflective materials combined with high-contrast or fluorescent colors supported safety and stimulation. This pattern suggests that when targeting emotional comfort and stress relief, designers should combine tactile softness with calming visual tones. Conversely, when emphasizing safety and vitality, pairing technical materials with bold, high-visibility colors proves more effective.

Pattern design serves as an important bridge between nature connection and cultural identity. Nature-inspired patterns directly support biophilic and aesthetic quality by evoking connection with natural environments, while geometric patterns incorporating cultural motifs can simultaneously express aesthetic quality, biophilic quality, and sociality. The success of Patagonia’s folk-inspired patterns and Cotopaxi’s regionally influenced designs demonstrates the potential of layering both pattern types to address multiple healing dimensions while maintaining cultural authenticity.

Finally, structural adaptability emerged as a key strategy for enhancing user autonomy. Adjustable components such as hoods, cuffs, waistbands, and ventilation zippers consistently aligned with independence, accessibility, pleasantness, and safety by allowing users to control their microenvironment based on activity intensity, weather conditions, and personal preference. The effective implementation by Arc’teryx and The North Face suggests that designers should incorporate adjustable elements in high-contact and ventilation zones while ensuring that adjustment mechanisms remain intuitive and aesthetically integrated rather than appearing as functional afterthoughts.

These practical applications demonstrate how healing design can extend beyond conceptual frameworks to offer realistic design strategies for outdoor fashion brands. By integrating ergonomic structure, tactile materiality, visual balance, and emotional resonance, designers can develop products that embody both functional performance and restorative wellbeing, aligning with contemporary needs for health-conscious and emotionally supportive fashion.

## 5 Conclusion

This study examined how outdoor fashion can serve as a theoretically grounded medium for expressing healing values that address psychological, physical, and social wellbeing. Drawing upon environmental psychology and healing design theory, the research developed a framework linking Healing Environments, Healing Design Characteristics, and Healing Effects, and applied it to the visual analysis of 805 products from six global outdoor brands.

The analysis identified how four core design elements—structure, pattern, material, and color—function as vehicles for healing expression. Structural features such as ergonomic, semi-open, and adjustable designs facilitate physical ease and adaptability, conceptually supporting healing design characteristics including pleasantness, independence, accessibility, openness, and safety. These characteristics, in turn, are theoretically associated with healing effects such as stability, joy, and tension relief. Pattern elements, including nature-inspired and geometric motifs, reflect biophilic and cultural qualities associated with familiarity and social connection. Soft and reflective materials were found to convey tactile comfort and perceived protection, while color strategies—particularly nature-inspired and soft tones—were theoretically linked to relaxation, visibility, and aesthetic quality.

When interpreted collectively, these elements suggest that outdoor fashion design may achieve stronger healing potential through integrative combinations rather than isolated features. Conceptual synergy between structure, material, and color appears to support multi-dimensional comfort, aesthetic harmony, and psychological stability. Although these relationships are interpretive rather than causal, they highlight the theoretical value of holistic, cross-sensory design approaches in promoting wellbeing through fashion.

Several limitations should be acknowledged. The study’s scope was limited to visual analysis of product images from six global brands and did not include direct user evaluation or physiological measurement. Therefore, the findings represent theoretically grounded associations rather than empirically verified outcomes. Additionally, the coding process, while validated through substantial inter-coder reliability (*κ* = 0.74–0.76) and expert consensus, involved interpretive judgment in linking design elements to Healing Design Characteristics. These limitations indicate that the findings should be interpreted as conceptual associations requiring empirical validation rather than established causal relationships.

As this research employed an exploratory qualitative approach, its primary goal was to conceptually map and interpret relationships rather than to statistically verify them. Accordingly, the empirical depth of the study lies in its systematic coding and expert validation processes rather than in quantitative inference. This interpretive focus aligns with the exploratory aim of identifying theoretical linkages among Healing Design Characteristics, design elements and Healing Effects, while future research is encouraged to validate these relationships through user-centered and data-driven studies.

Despite these limitations, this study offers practical value by providing guidelines for design strategies in outdoor fashion brands that reflect contemporary societal demand for healing and recovery. The systematized framework connecting Healing Environments, Healing Design Characteristics, and design elements can serve as a reference tool for designers and brand strategists seeking to integrate wellbeing considerations into product development. By clarifying how specific design elements—such as ergonomic structures, nature-inspired patterns, soft materials, and calming color palettes—may theoretically support healing experiences, this research provides actionable insights for commercial, community, and therapeutic contexts. The design strategies and theoretical associations derived from this study are expected to serve as foundations for future research and practical applications in outdoor fashion.

To validate and extend these findings, future studies should incorporate empirical research utilizing psychological response measurements and physiological data from actual users wearing these garments in real-world outdoor contexts. Additionally, follow-up investigations considering diverse cultural backgrounds, age groups, and usage contexts are necessary to understand how healing design elements are perceived and experienced across different populations. Longitudinal studies examining the sustained effects of healing-oriented outdoor fashion would further strengthen the theoretical framework established in this research. Such empirical validation would enable outdoor fashion brands to more confidently integrate healing values and wellbeing outcomes into their product development processes, marketing strategies, and broader health-promoting strategies.

## Data Availability

The original contributions presented in the study are included in the article/supplementary material, further inquiries can be directed to the corresponding author/s.
